# A Fast and High-Accuracy Foreign Object Detection Method for Belt Conveyor Coal Flow Images with Target Occlusion

**DOI:** 10.3390/s24165251

**Published:** 2024-08-14

**Authors:** Hongwei Fan, Jinpeng Liu, Xinshan Yan, Chao Zhang, Xiangang Cao, Qinghua Mao

**Affiliations:** 1 School of Mechanical Engineering, Xi’an University of Science and Technology, Xi’an 710054, China; hw_fan@xust.edu.cn (H.F.); 18894594581@163.com (X.Y.); zhangc2124@163.com (C.Z.); caoxg@xust.edu.cn (X.C.); maoqh@xust.edu.cn (Q.M.); 2 Shaanxi Key Laboratory of Mine Electromechanical Equipment Intelligent Detection and Control, Xi’an University of Science and Technology, Xi’an 710054, China

**Keywords:** belt conveyor, image recognition, target detection, deep learning, model compression

## Abstract

Foreign objects in coal flow easily cause damage to conveyor belts, and most foreign objects are often occluded, making them difficult to detect. Aiming at solving the problems of low accuracy and efficiency in the detection of occluded targets in a low-illumination and dust fog environment, an image detection method for foreign objects is proposed. Firstly, YOLOv5s back-end processing is optimized by soft non-maximum suppression to reduce the influence of dense objects. Secondly, SimOTA label allocation is used to reduce the influence of ambiguous samples under dense occlusion. Then, Slide Loss is used to excavate difficult samples, and Inner–SIoU is used to optimize the bounding box regression loss. Finally, Group–Taylor pruning is used to compress the model. The experimental results show that the proposed method has only 4.20 × 10^5^ parameters, a computational amount of 1.00 × 10^9^, a model size of 1.20 MB, and an mAP_0.5_ of up to 91.30% on the self-built dataset. The detection speed on the different computing devices is as high as 66.31, 41.90, and 33.03 FPS. This proves that the proposed method achieves fast and high-accuracy detection of multi-layer occluded coal flow foreign objects.

## 1. Introduction

The intelligentization of coal mines is the core technical requirement to support the high-quality development of the coal industry. As the key equipment of the main coal transportation system, the intelligentization of belt conveyors is required to guarantee the safe and efficient transportation of coal, which is of great significance to improving the safety level of coal mines and ensuring the stable supply of coal [1,2]. In the process of the continuous high-strength operation of coal transportation in the complex and harsh environment of coal mines, the materials conveyed by conveyor belts are often mixed with sharp foreign objects, such as gangues and iron wares, which easily cause local damage to conveyor belts, resulting in safety accidents. If foreign objects such as gangues and iron wares can be identified and sorted before the appearance of major safety hazards, the continuous, stable, green, and efficient production of coal mines will undoubtedly be guaranteed, thus advancing the intelligent development of coal mines [3,4].

Common methods for foreign object detection on the belt conveyors of coal mines include manual sorting, iron removal, spectral detection, and image recognition [5]. With the development of intelligent coal mines, inefficient and labor-intensive manual sorting methods cannot meet the requirements of intelligent and unmanned operation. The iron removal method cannot detect non-metallic foreign objects such as gangues and wood, and the efficiency of spectral detection is low. Compared to traditional image recognition algorithms, the target detection algorithm based on deep learning has received widespread attention due to its strong feature extraction ability, good generalization capability, and high recognition efficiency. At present, many scholars use machine vision and deep learning methods to conduct in-depth research on the intelligent identification of foreign objects such as gangues and iron wares on the conveyor belts of coal mines.

In an investigation of the influence of coal mine illumination on the recognition of foreign objects, Wang et al. [6] studied the changing rules of different illumination and gangue surface features. Li et al. [7] studied the influence of light, water, and dust on the image characteristics of gangues through gray and texture features. Cao et al. [8] studied a high-quality image acquisition method under multiple factors such as light source distribution and illumination, and based on this research, the recognition rate of gangues was improved. Li et al. [9] used the Retinex algorithm to adjust the overall brightness of the image and reduce the interference of dust, water mist, etc., and used the least squares support vector machine as a classifier to prove that enhancing the illumination can improve the recognition rate of gangues.

In the dust fog environment, Hao et al. [3] investigated dust interference and the high-speed operation of conveyors by introducing the Convolutional Block Attention Module (CBAM) into YOLOv5, replacing the ordinary convolution and loss function to improve the detection accuracy and speed of foreign objects. Mao et al. [10] examined the low foreign object detection accuracy caused by environmental factors such as dust fog and blurring by using a dark channel prior defogging algorithm to reduce the impact of dust fog on image clarity. Using 3 × 3 convolution to refine the image to reduce the blurring under high-speed conditions, the detection accuracy of foreign objects on the enhanced YOLOv5 algorithm was improved. In view of damage and interference to the conveyor belts’ surface in a low-illumination and dust fog environment, Fan et al. [11] enhanced the clarity of the image by using limited contrast adaptive histogram equalization and then improved YOLOv5, which enhances the detection effect of foreign objects. In an environment with a high concentration of dust and water mist, Fan et al. [12] proposed a method of the dust removal and illumination enhancement of the images based on dark channel segmentation and fusion; the enhanced image details were more obvious, and the detection effect of foreign objects was improved.

In terms of improving the performance of the target detection model, Gao et al. [13] used the CBAM to improve the MobileNetV3 network, reduced the overfitting of the model through data enhancement, and improved the accuracy of gangue detection. Xu et al. [14] identified gangue images based on the Convolutional Neural Network (CNN) and optimized the model with the model pruning method, which improved the detection accuracy and reduced the model size. Guo et al. [15] used the optimization method to improve the classical neural network and constructed a gangue detection model for small samples, which reduced the model size and improved the detection accuracy. Wu et al. [16] proposed a foreign object detection model based on the Faster R–CNN and bidirectional feature pyramid network, which improved the detection accuracy. Lv [17] used the VGG16 to improve the Faster R–CNN network to complete the intelligent identification of gangues and irons with a high recall rate. Hu et al. [18] used Focal Loss to replace the cross-entropy loss function of the YOLOv3 model and optimized its hyperparameters, identifying the anchor rods, irons, and nuts with high confidence. Ren et al. [19] proposed a foreign object detection method based on the improved CenterNet, which reduced false and missed detections. Wang et al. [20] optimized the SSD model with the deep separable convolution and generalized intersection over union (GIoU) loss function, which improved the detection accuracy and speed. Cheng et al. [21] proposed a lightweight network that integrated residual information, which improved the detection accuracy of foreign objects and detection speed. Chen [22] improved YOLOv5 and designed a foreign object detection system for conveyor belts, which realized the real-time detection of foreign objects. Based on the above studies, intelligent detection based on deep learning is the main technical means of foreign object detection, and measurable progress has been achieved.

However, in actual coal mines, foreign objects in real-time coal flow are often in a state of mutual occlusion. Ding [23] used a near-infrared camera and a visible light camera to form a binocular system, established a real-time multi-level fusion detection system for sensors, samples, and features, the SVM was combined with YOLOv3, and the length suppression algorithm was introduced to solve the problem of occlusion. Li et al. [24] used SKNet to replace MobileNetv3 as a backbone network and added a shallow detection structure and optimized loss with CIoU, which solved the problems of large memory occupation and low detection speed and accuracy for small and occluded targets, but the occlusion degree was low, and the categories of identified targets were few. Zhang et al. [25] designed a lightweight YOLOv4 model to solve the problems of image blurring and poor real-time performance at high speed; the dataset constructed by six categories of non-coal foreign objects contained a small number of occluded samples, and the occlusion degree was also low. In the process of using KinD++ and YOLOv4 to achieve the efficient detection of foreign objects in low illumination, Chen et al. [26] used the GridMask method to partially mask foreign object images, thereby expanding the image dataset. Mao et al. [10] conducted a test on YOLOv5, and the missed detection of foreign objects was reduced after model improvement, but there was also a low-occlusion case. Yao et al. [27] used iron, wood, and gangue targets to imitate foreign objects, and the mutual occlusion among foreign objects was considered, and data enhancement as random occlusion was used to expand the number of samples; high accuracy, recall, and mAP_0.5_ were obtained on the enhanced YOLO model, but the environmental factors were not considered.

In view of the above work, it is found that the detection of foreign objects in coal flow mainly focuses on the recognition of non-occluded foreign objects. Research on partially occluded foreign objects does not fully consider the influence of low illumination and dust fog. Constructed occluded samples are simple, the occlusion degree is low, and occluded object proportion is limited. Occlusion will cause a low detection accuracy of foreign objects. Therefore, considering the actual situation of coal mines, the foreign object target detection dataset with different occlusion degrees and categories in a low-illumination and dust fog environment will be constructed, and a fast and high-accuracy intelligent detection method for foreign objects considering complex occlusion cases will be proposed to solve the problem of the poor detection effect and weak real-time performance of occluded foreign objects.

## 2. Image Acquisition of Multi-Layer Occluded Foreign Objects

### 2.1. Experimental Platform and Parameters

**Figure 1:**
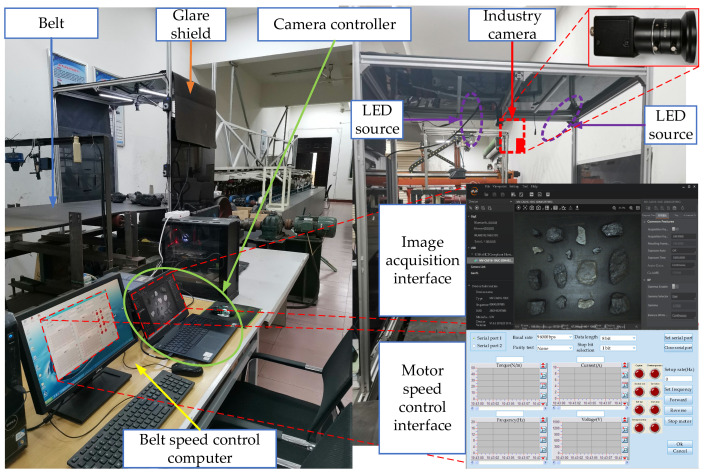
Image acquisition platform of coal flow foreign objects.

As shown in Figure 1, the image acquisition platform of coal flow foreign objects of belt conveyors is composed of a belt conveyor, belt speed control computer, industrial camera, camera controller, light source, and so on. The parameters are shown in Table 1. The conveyor belt is 11 m long and 0.8 m wide. The belt speed control computer controls the motor to realize the speed regulation of the belt conveyor. An industrial area array camera is selected, and its image resolution is 1440 × 1080 and acquisition frame rate is 160~190 FPS. The vertical distance from the camera mirror to the belt is 0.81 m. The camera controller is connected through USB. The light source and camera are arranged directly above the detection area.

In the experiment, the basic model of target detection is YOLOv5s, the input image size is 1440 × 1080, the batch size is 16, the epochs are 300, the initial learning rate is 0.01, and the SGD is used to optimize the loss function. The momentum factor and weight attenuation factor are 0.937 and 0.0005. The hardware and software configuration used for the model training is shown in Table 2. In order to verify the effectiveness of the model compression method, the GPU and CPU are used to compare the model detection speed, and their corresponding parameters are shown in Table 3.

### 2.2. Image Data Construction

Taking gangues, anchor rods, boasts, wires, nuts, and sticks as the detection objects, the different degrees of occlusion are set up, and the occluded images considering illuminance and belt speed are collected. Due to the difficulty of creating occluded images and the inability to simulate all occlusion conditions, the non-occluded images are combined with black rectangles to form a multi-layer occluded dataset with 690 real occluded images, for a total of 1134 images. The number of images used for the training, verification, and test is 827, 207, and 100, respectively. The occlusion degree includes no occlusion (0%), general occlusion (1~35%), severe occlusion (35~80%), and complete occlusion (≥80%). The occlusion type includes intra-class occlusion and inter-class occlusion.

Table 4 shows clear images of real and synthetic occluded images, with illuminations of about 80, 185, and 280 Lux. In order to more clearly display the target in the 80-Lux image, after it is transferred from RGB space to HSV space, the brightness of the V channel is enhanced by 3 times, which is represented by the 80–V5 case in Table 4.

It can be seen from Table 4 that under 80 Lux, the small nuts, wires, gangues, and other targets above the coal in real occluded images have low visibility and are all interfered with by the background. The anchor rods in the middle area are blocked by gangues and sticks. The visual area of the anchor rods is smaller, which is divided into three sections, resulting in information loss and positioning difficulty. The three irregular wires blocked by gangues in the lower right corner are blocked by each other, which is difficult to detect. The six categories of targets in the synthetic occlusion case are also disturbed by the background. Adding black rectangular boxes increases the loss of target information, maintains the difficulty of occlusion detection, and enriches the occlusion type. For 185 Lux, the six categories of targets are similar. The two sticks on the right are mixed in the gangue pile, making their features unclear. About half of the information on the nut target in the middle is lost, and the learning feature is lower. The objects in the synthesized image are truncated by long rectangular boxes, which increases positioning difficulty. The images under 280 Lux are less disturbed by the background; it can be clearly seen that the occluded objects are cut into several segments, but most of the occluded targets also lose a lot of information.

Table 5 shows real and synthetic obscured images considering blur and dust fog. The belt speed for the blurred images is about 2 m/s. In order to more intuitively obtain the dust fog concentration, the brightness of the V channel of the dust fog images is enhanced by 3 times, which is represented by the Dust fog–V3 case in Table 5.

**Figure 2:**
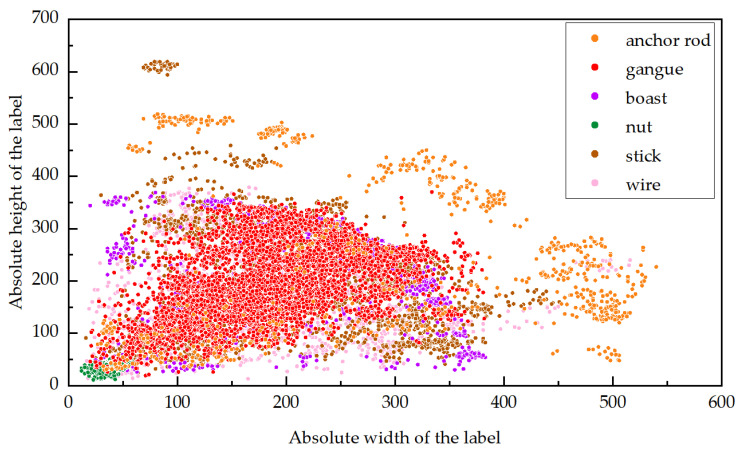
Absolute scale of foreign object.

**Figure 3:**
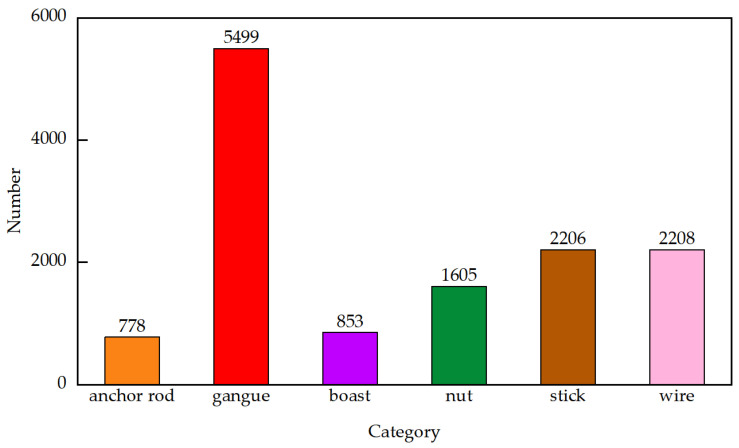
Foreign object number bar chart.

Figure 2 is a distribution map of all label sizes in the training set and verification set of multi-layer occluded coal flow foreign object images. The horizontal and vertical coordinates represent the absolute width and height of the label box. Figure 3 shows the number bar graph of the labeled categories in the training set and verification set. The horizontal and vertical coordinates represent the labeled categories and the corresponding number.

According to Figure 2 and Figure 3, it can be seen that a small number of anchor rods, sticks, gangues, boasts, wires, and all nuts in the occluded case are small targets; some nuts reach the minimum target, accounting for 12.21%. Combined with Table 4 and Table 5, it can be seen that due to occlusion, the actual width and height of most targets are far from the width and height of the 100~400-pixel size shown in Figure 2. The actual visualization area is small, but it is still larger than the visualization area of the nut.

In summary, the multi-layer occluded coal flow foreign object dataset constructed by using real and synthetic occlusion simulates a variety of cases. The number of data for each type of target is sufficient. It not only considers the factors such as illuminance, dust fog, and belt speed, but it also considers the occlusion degree and type, taking into account the dense degree at the same time. It can be seen that the constructed dataset has high complexity, a certain detection difficulty, and a more comprehensive design.

## 3. Detection Model of Multi-Layer Occluded Foreign Objects

### 3.1. YOLOv5s Network Model

**Figure 4:**
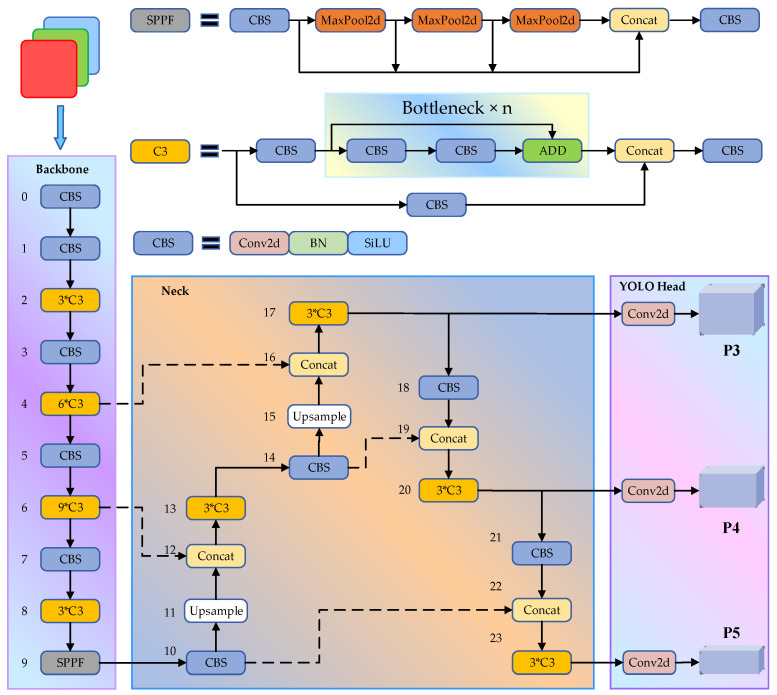
Foreign object width and height distribution map.

Considering model complexity, detection accuracy, and speed, the 7.0 version of YOLOv5s is used as a benchmark model, and it is optimized from the following two aspects: accuracy improvement for occluded object detection and speed improvement for an embedded system’s deployment. The former is mainly improved from the perspective of the model back-end processing algorithm, label allocation strategy, and loss function, while the latter uses model pruning to speed up the detection process. YOLOv5s consists of the Input, Backbone, Neck, and Head, as shown in Figure 4.

Input: This consists of mosaic data enhancement, adaptive image scaling, and adaptive anchor frame calculation. The objective of mosaic data enhancement is to randomly crop and scale images and then randomly splice them into one image. Adaptive image scaling consists of scaling images to a standard size that is accepted by the network. Adaptive anchor frame calculation refers to a process where the network outputs the predicted frame based on an initial anchor frame and compares it with the real frame, calculates the difference between the two and updates it in reverse, and then adjusts the network parameters.

Backbone: The backbone network of YOLOv5s mainly includes the Conv, C3, and SPPF modules, which are used to extract input image features. Conv consists of a convolution layer (Conv2d), a batch normalization layer (BN), and a SiLU activation function. The convolution layer is used to extract local spatial information, the normalization layer is used to normalize feature distribution, and the activation function is used to achieve nonlinear mapping. C3 consists of three standard convolutions and multiple bottleneck structures (Bottleneck), which are used to increase the depth and receptive field of the network and improve feature extraction capability. The SPPF passes the input through multiple maximum pooling layers (MaxPool) with different kernel sizes in parallel, and then concatenation (Concat) is performed to fuse different features, which solves the multi–scale issue of detected targets.

Neck: This is composed of a feature pyramid network (FPN) and path aggregation network (PAN). The fine-grained spatial information of the shallow feature map and the semantic information of the deep feature map are fused from top to bottom through the FPN, and the low-level positioning information is easily transmitted upward from the bottom to the top through the PAN. The combination of the two strengthens the network feature fusion ability. Upsample is used to increase the size of the feature map.

Head: This is used to output the detection results of the targets. The CIoU loss function is used to optimize the accuracy of the bounding box. The regression of the target box is described from three aspects: overlapping area, center point distance, and aspect ratio.

### 3.2. Soft Non-Maximum Suppression

The non-maximum suppression (NMS) is an important part of the object detection model, which is used to suppress a large number of redundant candidate detection boxes. In the case of target aggregation, occlusion, etc., in the model, it is easy to set the confidence score of the candidate box whose overlap degree is greater than the predetermined threshold to 0, resulting in the missed detection of adjacent targets. For the detection of occluded foreign objects, Soft–NMS [28] is used to optimize the YOLOv5s model. The flow of the Soft–NMS algorithm is shown in Algorithm 1.
**Algorithm 1:** Soft–NMS (Non-maximum suppression)
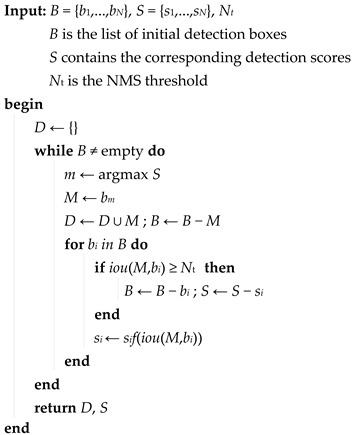


The Soft–NMS algorithm attenuates the confidence score of candidate boxes with an overlap greater than the threshold by the attenuation function instead of the direct suppression of the NMS algorithm, which can reduce the missed detection cases of the model. When the overlap of two boxes is greater, the degree of the confidence score attenuation of the corresponding candidate box is greater. The attenuation function includes a linear and a Gaussian weighted function, and the formulas are shown below.

(1) $$s_{i}=\left\{\begin{array}{ll} s_{i}, & iou(M,b_{i})<N_{t}\\ s_{i}(1-iou(M,b_{i})), & iou(M,b_{i})\geq N_{t} \end{array}\right.$$

(2) $$s_{i}=s_{i}e^{-\frac{iou(M,b_{i})}{\sigma_{1}}},\;\forall b_{i}\notin D$$

where $s_{i}$ is the confidence score of the *i*–th candidate box, *M* is the candidate box with the current maximum confidence, $b_{i}$ is the *i*–th candidate box, $iou(M,b_{i})$ is the ratio of the intersection and union of the area of the candidate box *M* and $b_{i}$, $N_{t}$ is the overlap threshold, D is the retained high-score detection box set, and the NMS and Soft–NMS overlap threshold uses a default value of 0.6. The value of the standard deviation $\sigma_{1}$ of the Gaussian function defaults to 0.5. The attenuation function in the Soft–NMS algorithm is the Gaussian weighted function.

### 3.3. SimOTA Label Allocation Optimization

Appropriate sample allocation can improve the model’s detection performance. YOLOv5 adopts a static sample allocation strategy based on shape matching. In an image with serious occlusion and dense targets, it is difficult to meet different detection scenarios by artificially setting hyperparameters, and it is easy to cause an imbalance between positive and negative samples. The optimal transport assignment (OTA) [29] strategy analyzes the sample allocation from a global perspective and converts it into an optimal transport (OT) problem, which reduces the negative impact of ambiguous samples and can adapt to multi-target overlaps and occlusion, small targets, etc. However, using the Sinkhorn–Knopp iterative method to solve the OT problem will lead to extra training time. The simplifying OTA (SimOTA) [30] method obtains an approximate solution of the OT problem through a dynamic top–k strategy, which can reduce the model training time.

The goal of the OT problem is to find an optimal transportation plan $\pi *=\{\pi_{i,j}\;|i=1,2,\ldots m;\;j=1,2,\ldots n\}$ so that all goods from the provider can be transported to the demander at the minimum cost.

For a target detection task, the cost matrix C is composed of classification and regression loss. If there are *m* ground truth boxes (GT) and *n* anchor boxes (Anchor) in the image, each GT is regarded as a provider with *k* ($p_{i}=k,\;i=1,2,\ldots m$) positive samples, and each Anchor that needs a unit sample is regarded as a demander. The cost of transporting a unit of positive samples from $gt_{i}$ to Anchor $a_{j}$ is defined as the weighted sum of classification and regression loss.

(3) $$c_{ij}^{fg}=L_{cls}(P_{j}^{cls},G_{i}^{cls})+\alpha L_{reg}(P_{j}^{box},G_{i}^{box})$$

where $P_{j}^{cls}$ and $P_{j}^{box}$ are the predicted classification score and bounding box, $G_{i}^{cls}$ and $G_{i}^{box}$ are the GT classification score and bounding box, $L_{cls}$ and $L_{reg}$ are the cross-entropy loss and IoU loss, and α is the value of a balance coefficient; the default value is 3.

Because the Anchor can be assigned not only as a positive sample but also as a negative sample, another provider (background) is introduced to provide n−m×k negative samples. The cost of transporting a unit of negative samples from the background to the Anchor is $c_{ij}^{bg}$.

(4) $$c_{ij}^{bg}=L_{cls}(P_{j}^{cls},\emptyset )$$

where ∅ is the background.

The last row of $C^{fg}\in R^{m\times n}$ is spliced with $C^{bg}\in R^{1\times n}$ to form a cost matrix C of (m+1)×n. The updating provider vector formula is shown below.

(5) $$p_{i}=\left\{\begin{array}{ll} k, & if\;i\leq m\\ n-m\times k, & if\;i=m+1 \end{array}\right.$$

**Figure 5:**
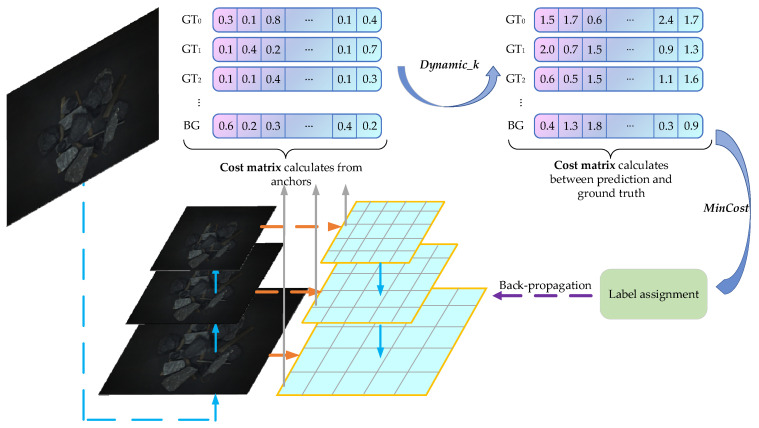
Logic flow of SimOTA.

After obtaining the cost matrix C, it is necessary to determine the number dynamic_k of positive samples for each GT. The calculation method for the dynamic_k value consists of calculating the IoU between the current GT and all prediction boxes generated by the Anchor, selecting the top 10 largest samples, and then rounding down the sum of the 10 IoU values, which is the dynamic_k; the minimum number of positive samples is guaranteed to be 1. After obtaining the positive samples, the cost matrix is traversed, and the first dynamic_k samples with the smallest loss for each GT are taken as positive samples. Finally, the situation where one prediction frame is assigned to multiple GTs is eliminated, and the division of positive and negative samples is completed. A logic diagram of the SimOTA algorithm is shown in Figure 5.

Through the SimOTA label allocation strategy, more reasonable samples are allocated for dense, occluded, and small targets from a global perspective, reducing missed detection caused by ambiguous samples and improving the detection performance of the model.

### 3.4. Improvement of Loss Function

#### 3.4.1. Slide Loss

For a single-stage detector, many bounding boxes are not iteratively filtered out, resulting in a large number of simple samples and sparse difficult samples, which leads to sample imbalance. During the training process, the cumulative contributions of simple samples dominate the updating of the model, resulting in model overfitting. YOLOv5 uses the cross-entropy loss function to calculate classification and target loss and uses CIoU to calculate the positioning loss. In order to excavate difficult samples and assign higher weights to difficult samples, Slide Loss [31] is used to optimize the cross-entropy function. Setting high weights near μ will increase the relative loss of difficult samples and focus more on difficult and misclassified samples. The formula for the Slide Loss is as follows.

(6) $$f(x)=\left\{\begin{array}{ll} 1, & x\leq \mu -0.1\\ e^{1-\mu }, & \mu -0.1<x<\mu \\ e^{1-x}, & x\geq \mu \end{array}\right.$$

where μ is the average value of IoU calculated by all bounding boxes, those less than μ are negative samples, and those greater than μ are positive samples.

**Figure 6:**
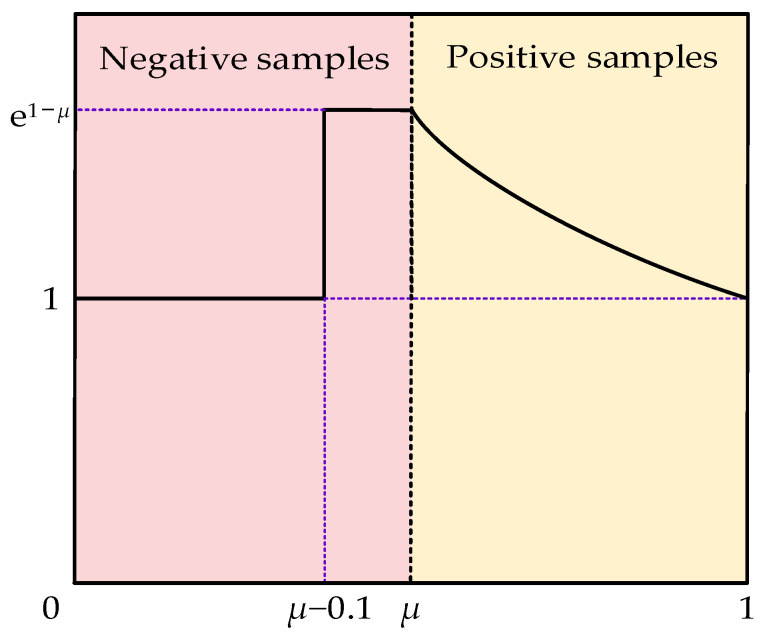
Schematic diagram of Slide Loss.

A schematic diagram of Slide Loss is shown in Figure 6. The samples near the boundary often suffer large loss due to unclear classification. If the network can make full use of the difficult samples near the μ value, the detection performance of the model can be improved. However, the number of such samples is rare, so assigning high weights to such difficult samples increases the relative loss, making the network pay more attention to difficult and misclassified samples.

#### 3.4.2. Inner–SIoU

The traditional IoU loss function depends on geometric factors such as the overlap area, distance, and aspect ratio between the prediction box and GT, but it does not consider the direction between them, resulting in a poor detection effect and it being unable to adaptively adjust. The original YOLOv5s adopts the CIoU loss function without considering the angle factor. This article uses SIoU with Inner–IoU [32] to build Inner–IoU to calculate IoU loss. Compared to the traditional loss function, Inner–SIoU not only considers the factors such as the overlapping area, distance, angle, and direction, but it also controls the scale of the auxiliary frame by adjusting the scale factor ratio to adapt to different IoU samples, improving the detection accuracy of small and occluded targets.

**Figure 7:**
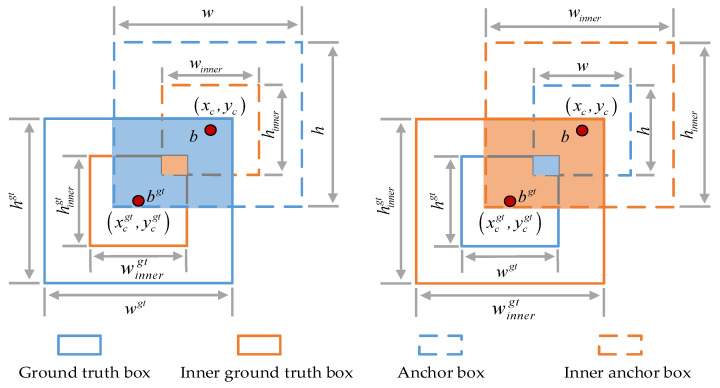
Schematic diagram of Inner–IoU.

**Figure 8:**
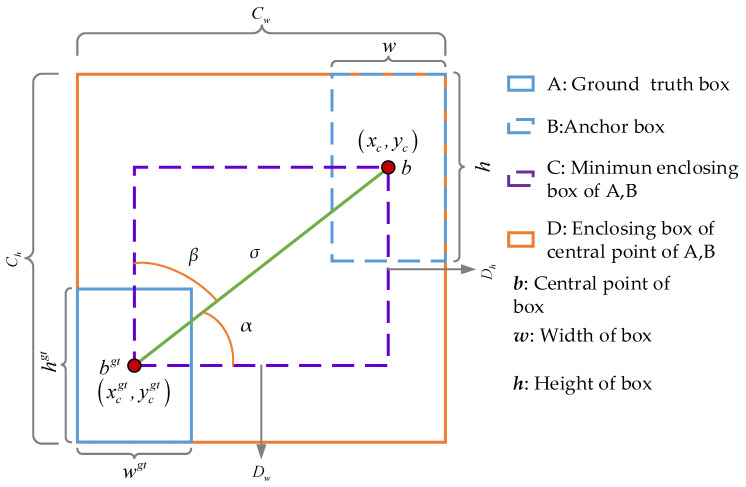
Schematic diagram of SIoU.

The formula of Inner–SIoU is shown in Equation (7).

(7) $$\begin{array}{cc} L_{Inner-SIoU} & =L_{SIoU}+IoU-IoU^{inner}\\ & =1-IoU+\frac{\Delta +\Omega }{2}+IoU-IoU^{inner}\\ & =1-IoU^{inner}+\frac{\Delta +\Omega }{2} \end{array}$$

where $IoU^{inner}$ is the Inner–IoU value, $L_{SIoU}$ is the SIoU loss, Δ and Ω are the distance and shape loss in SIoU, and Δ and Ω are shown in Equations (15) and (24). A schematic diagram of Inner–IoU is shown in Figure 7, the formula of Inner–IoU is shown in Equation (8), and a schematic diagram of SIoU is shown in Figure 8.

(8) $$IoU^{inner}=\frac{Inner_{inter}}{Inner_{union}}$$

(9) $$Inner_{inter}=\left(\min\left(b_{r}^{gt},b_{r}\right)-\max\left(b_{l}^{gt},b_{l}\right)\right)\times \left(\min\left(b_{r}^{gt},b_{r}\right)-\max\left(b_{l}^{gt},b_{l}\right)\right)$$

(10) $$Inner_{union}=\left(w^{gt}\times h^{gt}\right)\times {\left(ratio\right)}^{2}+\left(w\times h\right)\times {\left(ratio\right)}^{2}-Inner_{inter}$$

(11) $$b_{l}^{gt}=x_{c}^{gt}-\frac{w^{gt}\times ratio}{2},\;b_{r}^{gt}=x_{c}^{gt}+\frac{w^{gt}\times ratio}{2}$$

(12) $$b_{t}^{gt}=y_{c}^{gt}-\frac{h^{gt}\times ratio}{2},\;b_{b}^{gt}=y_{c}^{gt}+\frac{h^{gt}\times ratio}{2}$$

(13) $$b_{l}=x_{c}-\frac{w\times ratio}{2},\;b_{r}=x_{c}+\frac{w\times ratio}{2}$$

(14) $$b_{t}=y_{c}-\frac{h\times ratio}{2},\;b_{b}=y_{c}+\frac{h\times ratio}{2}$$

where $Inner_{inter}$ and $Inner_{union}$ are the intersection and union of the auxiliary bounding box of the target ground truth box (Inner ground truth box) and the auxiliary bounding box of the anchor box (Inner anchor box), $b_{l}^{gt}$, $b_{r}^{gt}$, $b_{t}^{gt}$, $b_{b}^{gt}$, and $b_{l}$, $b_{r}$, $b_{t}$, and $b_{b}$ are the horizontal and vertical coordinates of the left, right, upper, and lower sides of the Inner ground truth box and Inner anchor box. ($x_{c}^{gt},y_{c}^{gt}$), $w^{gt}$, $h^{gt}$, and ($x_{c},y_{c}$), w, and h are the midpoint coordinates, width, and height of the GT and Anchor. The ratio is the scale factor that controls the width and height of the auxiliary bounding box, and its value range is [0.5, 1.5]. When ratio<1, the auxiliary bounding box is shown on the left side of Figure 4. When ratio>1, the auxiliary bounding box is shown on the right side of Figure 4. When ratio=1, the auxiliary bounding box is consistent with the GT and Anchor. When ratio=1.10, a better balance can be achieved between the different categories of targets, and the detection effect is the best. The larger auxiliary frame can not only include more information, facilitating the utilization of contextual information, but also reduce error fluctuations caused by pixel deviation and improve model detection accuracy.

(15) $$\Delta =\sum_{t=x,y}\left(1-e^{-\gamma \rho_{t}}\right)$$

(16) $$\rho_{x}={\left(\frac{d_{w}}{c_{w}}\right)}^{2},\rho_{y}={\left(\frac{d_{h}}{c_{h}}\right)}^{2},\gamma =2-\Lambda$$

(17) $$d_{w}=\max\left(x_{c}^{gt},x_{c}\right)-\min\left(x_{c}^{gt},x_{c}\right)$$

(18) $$d_{h}=\max\left(y_{c}^{gt},y_{c}\right)-\min\left(y_{c}^{gt},y_{c}\right)$$

(19) $$c_{w}=\max\left(x_{c}^{gt}+\frac{w^{gt}}{2},x_{c}+\frac{w}{2}\right)-\min\left(x_{c}^{gt}-\frac{w^{gt}}{2},x_{c}-\frac{w}{2}\right)$$

(20) $$c_{h}=\max\left(y_{c}^{gt}+\frac{h^{gt}}{2},y_{c}+\frac{h}{2}\right)-\min\left(y_{c}^{gt}-\frac{h^{gt}}{2},y_{c}-\frac{h}{2}\right)$$

(21) $$\Lambda =1-2\times \sin^{2}\left(\arcsin\left(x\right)-\frac{\pi }{4}\right)=\sin\left(2\alpha \right)$$

(22) $$x=\frac{d_{h}}{\sigma_{2}}=\sin\left(\alpha \right)$$

(23) $$\sigma_{2}=\sqrt{d_{h}^{2}+d_{w}^{2}}$$

where $d_{w}$ and $d_{h}$ are the horizontal and vertical distances between the centers of GT and Anchor, $c_{w}$ and $c_{h}$ are the width and height of the minimum external rectangular frame of GT and Anchor, Λ is the angle cost, and its value range is [0, 1]; α and β are the angles between the line connecting the center point of GT and Anchor and the horizontal axis and plumb axis, and $\sigma_{2}$ is the distance between GT and the Anchor center point.

(24) $$\Omega ={\sum_{t=w,h}\left(1-e^{-w_{t}}\right)}^{\theta }$$

(25) $$\omega_{w}=\frac{\left|w-w^{gt}\right|}{\max\left(w,w^{gt}\right)},\;\omega_{h}=\frac{\left|h-h^{gt}\right|}{\max\left(h,h^{gt}\right)}$$

where θ is the degree of the attention to shape loss, its value range is [2, 6], and the default value is 4.

In the case of limited samples, Slide Loss can fully excavate difficult sample information and improve information utilization. Inner–SIoU not only considers the angle factor but also uses auxiliary border training to adapt to IoU samples of different scale sizes, which can improve the detection accuracy of small and occluded targets.

### 3.5. Model Pruning

The model compression method can be used to remove redundant parameters in a network, reduce the model complexity, and improve the detection speed. Group–Taylor [33] channel pruning in the structured pruning method can be used to prune the model. The square of the final loss change caused by deleting a single parameter is used as the parameter importance evaluation criterion, and the first-order Taylor expansion is used to approximately estimate the importance of a single parameter. The least important parameters are deleted until the preset global pruning rate is satisfied. Finally, fine-tuning is performed to partially restore the model accuracy. The formula for calculating the importance of a single parameter is shown in Equation (26).

(26) $$I_{z}^{\left(1\right)}(W)={\left(g_{z}w_{z}\right)}^{2}$$

(27) $$g_{z}=\frac{\partial E}{\partial w_{z}}$$

where W is the neural network parameter, $W=\{w_{0},w_{1},\ldots ,w_{z}\}$, $g_{z}$ is the gradient of the parameter, *z* is a positive integer with a value greater than 0, and E is the loss of the network.

The formula of the joint importance of a set of structural parameters $W_{SP}$ is shown in Equation (28).

(28) $$I_{SP}^{\left(1\right)}(W_{SP})≜\sum_{sp\in SP}I_{SP}^{\left(1\right)}(W_{SP})=\sum_{sp\in SP}{\left(g_{sp}w_{sp}\right)}^{2}$$

where SP is a non-empty set composed of all parameters in the convolution kernel and other structures, and sp is the element in the set SP.

By using the first-order Taylor expansion to approximate the importance of parameters, the model is pruned without the layer-by-layer parameter sensitivity analysis, and the gradient in the back-propagation process can be used in parallel to reduce the computational complexity and improve the detection speed.

## 4. Experimental Results and Analysis

### 4.1. Model Evaluation Indexes

The mean Average Precision (mAP) with IOU thresholds of 0.5, 0.75, and 0.5:0.95 is used to evaluate the detection accuracy of the model, which is recorded as mAP_0.5_, mAP_0.75_, and mAP_0.5:0.95_, respectively. The model complexity is evaluated by parameters, the model size, and floating point operations per second (FLOPs). The detection speed is measured by frames per second (FPS). The ratio of the number of parameters before and after model pruning is used to measure the model compression degree. The calculation of the mAP is as follows.

(29) $$P=\frac{\mathrm{TP}}{\mathrm{TP}+\mathrm{FP}}\times 100\%$$

(30) $$R=\frac{\mathrm{TP}}{\mathrm{TP}+\mathrm{FN}}\times 100\%$$

(31) $$\mathrm{AP}=\int_{0}^{1}P(R)d\left(R\right)$$

(32) $$m\mathrm{AP}=\frac{\sum_{i}^{N}AP_{i}}{N}\times 100\%$$

(33) $$CD=\frac{Parameters_{a}}{Parameters_{b}}$$

where TP, FP, and FN are the number of correct detection boxes, false detection boxes, and missed detection boxes, N is the number of categories, P and R are Precision and Recall, and AP is Average Precision (AP). $Parameters_{a}$ and $Parameters_{b}$ are the parameters of the model before and after pruning, and CD is the compression degree of the model.

### 4.2. Ablation Experiment of Accuracy Improvement Model

In order to verify the effectiveness of the Soft–NMS algorithm, SimOTA label allocation strategy, Slide Loss, and Inner–SIoU function, the ablation experiment is carried out under the same conditions on the above self-made occluded dataset based on the 7.0 version of YOLOv5s, and the influence of each improvement point on the model performance is evaluated. The results of the ablation experiment are shown in Table 6. Since this work mainly solves the problem of target occlusion from the loss function and does not modify the network structure, the accuracy of the model is mainly discussed here.

It can be seen from Table 6 that after the back-end processing algorithm is changed to Soft–NMS in Model 2, mAP_0.75_ and mAP_0.5:0.95_ increase greatly, both reaching 3.5%, and mAP_0.5_ increases by 0.3%, which proves that Soft–NMS plays a large role in dealing with dense and occluded targets. Based on Model 2, Model 3 enhances label the allocation strategy to SimOTA, and mAP_0.5_, mAP_0.75_, and mAP_0.5:0.95_ have great enhancement, with increases of 1.1%, 1.9%, and 2.1%, which shows that SimOTA has a significant effect on dense and occluded targets. On the basis of Model 3, Model 4 uses Slide Loss, mAP_0.5_ and mAP_0.75_ are increased by 0.6% and 0.5%, and mAP_0.5:0.95_ is reduced by 0.1%, which indicates normal fluctuation. On the basis of Model 4, Inner–SIoU is used in Model 5. When the growth potential of the model is about to reach its peak, mAP_0.5_, mAP_0.75_, and mAP_0.5:0.95_ are increased by 0.1%, 0.3%, and 0.3%. In summary, introducing the four modules in Table 6 is effective. The mAP_0.5_, mAP_0.75_, and mAP_0.5:0.95_ of the enhanced model are 2.1%, 6.2%, and 5.8% higher than the original YOLOv5s.

**Figure 9:**
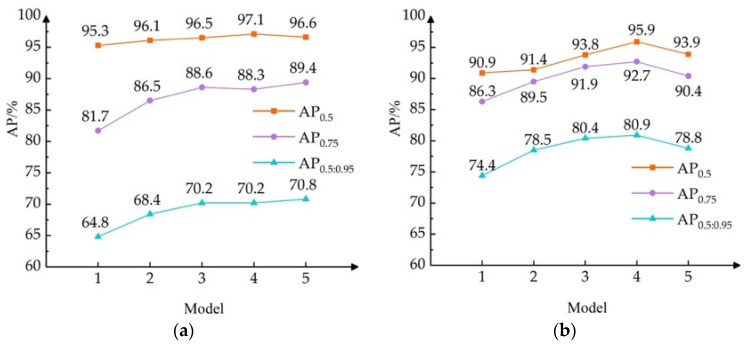

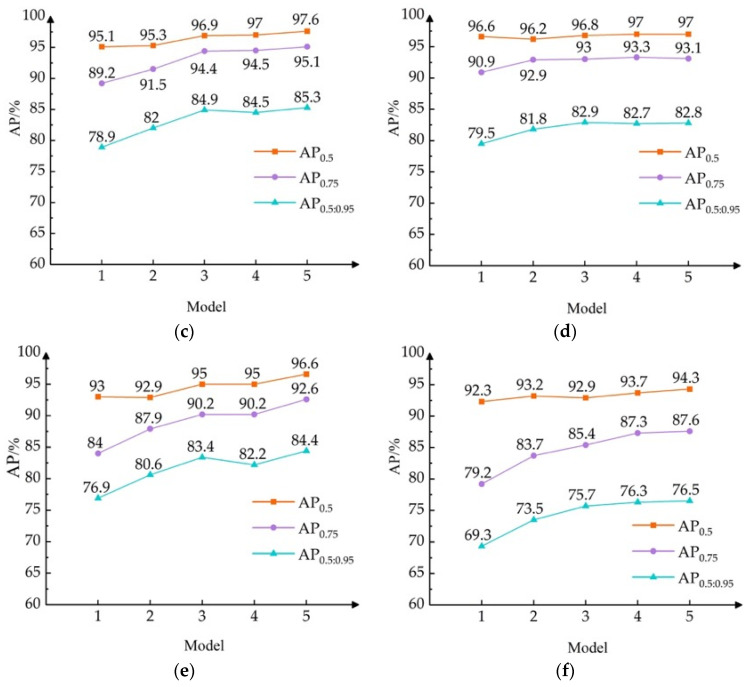
Enhancement effect of each improvement module on different categories: (**a**) nut; (**b**) anchor rod; (**c**) stick; (**d**) anchor rod; (**e**) boast; and (**f**) wire.

In order to evaluate the effect more objectively, the AP line charts of the six categories of targets are shown in Figure 9.

It can be seen from Figure 9 that the AP_0.5_, AP_0.75_, and AP_0.5:0.95_ of the six categories of targets are generally rising. The AP_0.5_ polylines of nuts, gangues, and wires are relatively flat, and the changes in the remaining targets are obvious. Aside from the fact that the polylines of the AP_0.75_ and AP_0.5:0.95_ of gangues are relatively flat, the AP_0.75_ and AP_0.5:0.95_ of other targets are greatly improved. Compared to AP_0.75_ and AP_0.5:0.95_, AP_0.5_ has lower positioning accuracy requirements. When the overlap between the prediction frame and real frame reaches 50%, the target is considered to be detected. The same prediction frame is more likely to be judged as the correct detection frame. The more frames that the model determines to be correctly detected, the higher the AP is, but the corresponding AP_0.5_ improvement is limited. On the basis of Model 1, Soft–NMS is used. The AP_0.75_ and AP_0.5:0.95_ of the six categories of targets change greatly; the increases are above 2% and 2.3%, respectively. Among these, the AP_0.75_ and AP_0.5:0.95_ of the nuts increase by 4.8% and 3.6%, which verifies that Soft–NMS has a significant effect on dealing with small and occluded targets. Then, SimOTA is changed in Model 2. The AP_0.75_ and AP_0.5:0.95_ of the gangues have the smallest increase, but the stick has the largest increase; both indicators increase by 2.9%. The increases in the remaining targets are still large, which verifies the strength of SimOTA in dealing with small and occluded targets. After adding Slide Loss to Model 3, the improvements in the six categories of targets increase and decrease, but only the nuts are decreased by 0.3% on AP_0.5_, and the rest are improved, with the highest increase of 2.9%. For AP_0.5:0.95_, it is mostly decreased, and anchor rods and wires are increased by 0.5% and 0.6%, which still indicates the effectiveness of Slide Loss. Finally, the addition of Inner–SIoU has a negative impact on the detection of anchor rods. Its AP_0.5_ decreases by 2.3%, and AP_0.5:0.95_ decreases by 2.1%. However, it has a gain effect on other targets. The gain effect of boasts is the most obvious, reaching 2.4% and 2.2%. The nuts also have an increase of 1.1% and 0.6%, which proves that Inner–SIoU can deal with different scale and shape targets.

### 4.3. Comparative Experiment of Model Compression Method

Model compression is studied while maintaining mAP_0.5_ > 90%. Based on the above Model 5, the main pruning methods used for structured pruning are compared based on model accuracy, complexity, and speed. Table 7 shows the results of different pruning methods, ‘+Random’ indicates that the Random pruning method is used on the basis of Model 5, and the compression degree is the numerical calculation result of parameters in the table.

It can be seen from Table 7 that the Group–Taylor and Group–Hessian pruning methods are significantly better than the Random, Lamp, and L1 methods. When the parameters, FLOPs, and model size of the first two methods are smaller than the latter three methods, mAP_0.5_ is still higher than the latter three methods. The higher the sparsity of the model, the more serious the accuracy loss. After the model parameters are compressed to about 1/17~1/14 of the original, the mAP_0.5_, mAP_0.75_, and mAP_0.5:0.95_ indexes are greatly reduced. After using the Group–Hessian and Group–Taylor methods, the parameters, FLOPs, and model size are reduced by 94.03%, 93.67%, and 91.67%, but mAP_0.5_ still maintains 91.30%.

**Figure 10:**
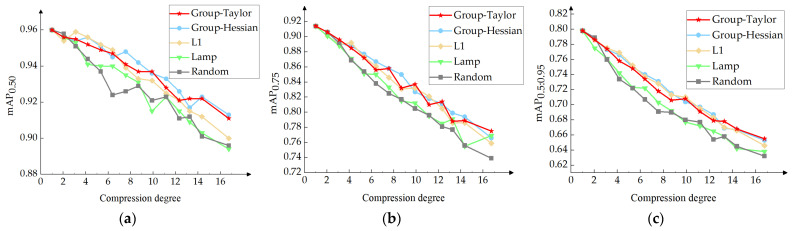
Model compression–accuracy line chart. (**a**) mAP_0.5_; (**b**) mAP_0.75_; (**c**) mAP_0.5:0.95_.

In order to more objectively show the difference in the detection accuracy of five pruning methods under different compression degrees, a line chart of mAP_0.5_, mAP_0.75_, and mAP_0.5:0.95_ is drawn, as shown in Figure 10. On the mAP_0.5_ chart, the accuracy values of the Group–Taylor and Group–Hessian methods are significantly better than those of the other three methods, and in particular, when the compression degree is close to 17, the difference is obvious. In the mAP_0.75_ and mAP_0.5:0.95_ chart, the accuracy values of the L1, Group–Taylor, and Group–Hessian methods are basically higher than those of the Random and Lamp methods. From the three charts, the Group–Taylor and Group–Hessian pruning methods are better.

In order to verify the superiority of the Group–Taylor method, the GPU and CPU devices are used to compare the detection speed of different pruning methods in Table 7. The results are shown in Table 8. The numerical value is the mean value of 100 images for five tests, and the batch size is uniformly 1.

It can be seen from Table 8 that on RTX 4080, the detection speed of the model before and after pruning is comparable. The five pruning methods can reach more than 100 FPS, but the difference is not obvious. On GTX 1050Ti, there is a significant difference in the model speed after and before pruning. The speed after pruning is 60 FPS, while the Group–Taylor method is the fastest, up to 66.31 FPS, which is 29.48 FPS higher than Model 5. The speed of Model 5 on GT 1030 is only about 40% of GTX 1050Ti. The model speed after pruning is about 2.58 to 2.90 times that before pruning; Group–Taylor is still the fastest. On Intel (R) Core (TM) i5–11400F, the Group–Taylor method is 4.32 times faster than the speed of Model 5, which meets the real–time processing requirements. From Table 7 and Table 8 and Figure 10, the superiority of the Group–Taylor method is verified.

**Figure 11:**
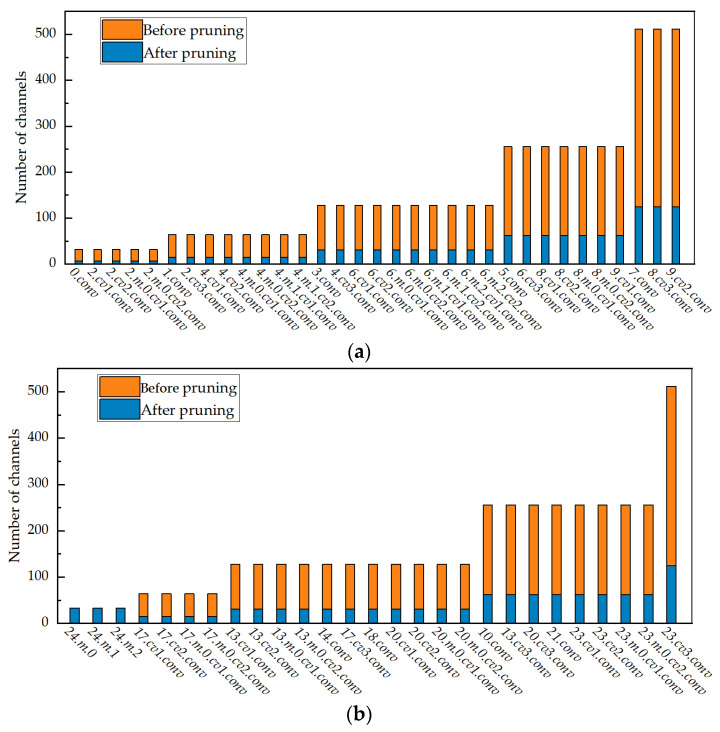
Comparison of number of convolution channels before and after pruning. (**a**) Backbone; (**b**) Neck + Head.

Figure 11 shows a comparison histogram of the number of convolutional layer channels in the Backbone, Neck, and Head before and after pruning, using the Group–Taylor method with a compression degree of 16.74 in the enhanced Model 5. In Figure 11a,b, the missed 11, 15,12, 16, 19, and 22 in the abscissa represent the Upsample and Concat modules that do not contain the convolution layer. The rest of the numbers represent the number of network layers, which corresponds to the number of layers in Figure 1. The number 24 represents the detection layer structure without pruning, and the number of channels in the three detection layers is 33. The ordinate represents the number of channels. Aside from the detection layer structure represented by 24, the number of channels before and after pruning in Figure 11 is arranged from low to high, i.e., [32, 64, 128, 256, 512], [7, 15, 31, 62, 125], and the numerical values correspond to the height of the histogram. From Figure 11, the number of channels in the 0~23 layers of the convolutional module after pruning is less than one-quarter of that before pruning. For example, the number of channels in the convolutional layer with 512 channels is 125 after pruning. Thus, the number of channels is greatly reduced, and the model is more compact.

The Group–Taylor pruning method with a compression degree of 16.74 is used to prune the improved model after optimizing the post-processing, label allocation strategy, Slide Loss, and Inner–SIoU loss function. For the high-accuracy detection model with only 4.20 × 10^5^ parameters, 1.00 × 10^9^ FLOPs, and 1.20 MB, an mAP_0.5_ of 91.30% is obtained, which is that of the final model. The model has detection speeds of 66.31, 41.90, and 33.03 FPS on the GTX 1050Ti, GT 1030, and CPU devices, which are far beyond those of the YOLOv5s, and achieves high real–time processing speed.

### 4.4. Comparative Experiment of Different Detection Algorithms

Under the same experimental conditions, YOLOv5n, YOLOv5n–Group–Taylor, YOLOv5s, YOLOv5m, YOLOv7, YOLOv7–X, YOLOv8n, YOLOv8s, and YOLOv8m are selected for comparison with the proposed method. Among them, YOLOv5n–Group–Taylor represents the pruning of YOLOv5n by the Group–Taylor method, which still needs to meet mAP_0.5_ > 90%. mAP_0.5_ is used as the accuracy evaluation index, and the model parameters, FLOPs, and size are used as the complexity evaluation indexes. The FPS calculated on the CPU is used to evaluate the detection speed. The results are shown in Table 9.

From Table 9, it can be seen that YOLOv5s can better balance the detection accuracy and speed in different versions. Using YOLOv7, the accuracy improvement is limited, and the FLOPs are as high as 103.20 × 10^9^, which is much higher than other algorithms. The mAP_0.5_ of YOLOv7–X is only 0.1% higher than that of YOLOv7, but its parameters, FLOPs, and model size are 1.94, 1.82, and 1.90 times than those of YOLOv7, respectively, and its detection speed is slower. YOLOv8m has the highest accuracy among these algorithms, but its detection speed is very low. Also using Group–Taylor pruning, the accuracy, complexity, and speed of YOLOv5n–Group–Taylor are not as good as the proposed method. Although the mAP_0.5_ of the proposed method is the lowest, it is still higher than 90%, and the parameters, FLOPs, and model size are far lower than other algorithms. More importantly, its detection speed is the fastest of the above algorithms. It is verified that the proposed method can realize the fast detection of occluded objects with high accuracy and low complexity on PC devices.

### 4.5. Visual Analysis of Detection Results in Different Scenarios

**Figure 12:**
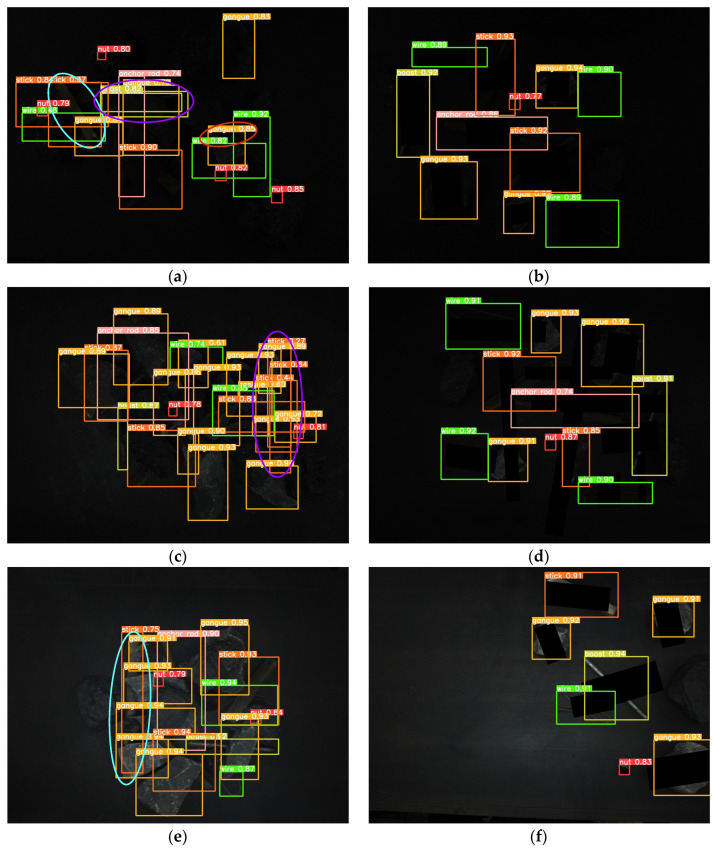
Image detection results under different illumination values: (**a**) 80-Lux real occluded image; (**b**) 80-Lux synthetic occluded image; (**c**) 185-Lux real occluded image; (**d**) 185-Lux synthetic occluded image; (**e**) 280-Lux real occluded image; and (**f**) 280-Lux synthetic occluded image.

In order to test the detection effect of the proposed method in a low-illumination environment, real and synthetic images under different illumination cases are tested. The visual results are shown in Figure 12.

From Figure 12, it can be seen that in the 80-Lux real occluded image (a), due to too low illumination, irregular wires blocked by the gangues have low visibility and a small visual area. In addition, due to the influence of nearby wires, the model misses a wire target. In the middle of the image, because the boast is blocked by the gangue and anchor rod, its contour is unclear, and there are multiple detection times and inaccurate positioning. On the left side of the image, two sticks are crossed, one stick is cut off by the coal and nut, which leads to a big positioning error when the model detects the other sticks, and the prediction frame is obviously larger. In the 185-Lux real occluded image (c), the influence of illumination on the occluded targets is significantly reduced. For the right long stick, it is truncated and occluded, resulting in repeated detection, the positioning error of the prediction box is big, and the confidence of the relatively accurate prediction box is only 0.64. In the 280-Lux real occluded image (e), it can be seen that the long stick on the left side is cut into several sections by gangues. The lower part of the stick is similar to the gangue, and the prediction frame is not positioned accurately. Most of the occluded targets in the real occluded images (a), (c), and (e) are effectively detected. Compared with the real occluded images, most targets in the 80-, 185-, and 280-Lux synthetic images (b), (d), and (f) have better detection results and higher confidence. It can be seen that the low-illumination environment has an impact on the detection of occluded targets, and this impact gradually decreases as the illumination increases.

**Figure 13:**
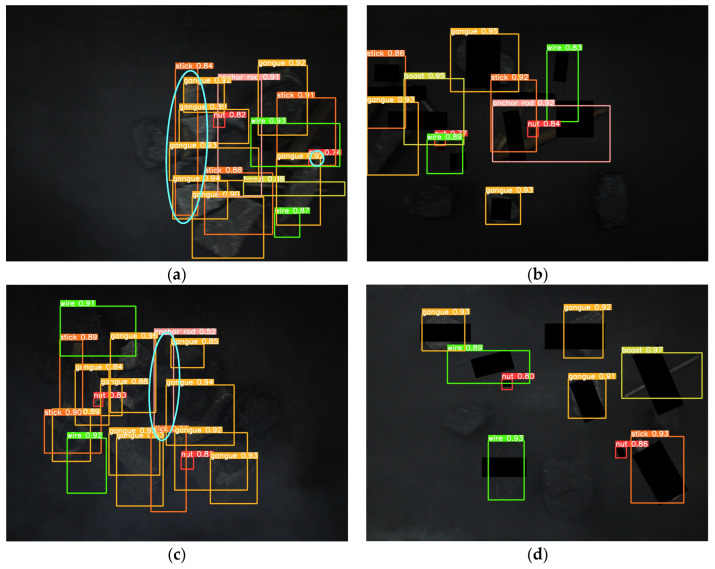
Occluded blur and dust fog image detection results. (**a**) Real occluded blur image; (**b**) synthetic occluded blur image; (**c**) real occluded dust fog image; (**d**) synthetic occluded dust fog image.

Figure 13 shows the visual results of the proposed method on the real and synthetic occluded images in a low-blur and high-dust fog environment.

Figure 13a,b were collected at a belt speed of 2 m/s. Subjectively, the overall ambiguity of the image is low, and the impact on large targets is limited. In Figure 13a, the small nut target on the right has low discrimination with the gangue contour, and the detection confidence of the nut is only 0.74, which affects the overall detection effect. Due to the occlusion of multiple pieces of gangues, the long stick on the right side is cut into multiple segments, and the model attention to the boundary is relatively insufficient, resulting in the poor positioning of the stick and obvious excess blanks at the upper end of the prediction frame, which does not contain the target component. The targets in Figure 13b are relatively sparse, and the overall detection effect is good. The dust fog images (c) and (d) were collected under a certain concentration. The concentration of dust fog is limited and has relatively little impact on the targets. The anchor rod in the middle of Figure 13c is obscured by the gangues and dust fog, resulting in unclear boundaries. The prediction frame does not fully include the anchor rod, and the detection effect is poor, with a confidence of only 0.52. The overall detection effect of the targets in Figure 13d is good. It can be seen that the factors such as blur and dust fog increase the difficulty of target detection, but the proposed method effectively detects most targets.

For the synthetic occluded images in Figure 12 and Figure 13, the occlusion type is background occlusion, there is a certain difference in color between the black rectangular block and the targets, and the targets are relatively sparse. The model can learn the difference between the two very well. However, occlusion results in there being a small learnable area, which affects the target detection effect. In terms of the sparseness, the detection effect of targets in a dense occlusion is much lower than that of targets in a sparse occlusion, which shows that the density of occluded targets greatly affects the detection effect.

Judging from the detection effect of each category of targets, small nut targets are seriously affected by occlusion, and missed detection, repeated detection, and poor positioning are mainly due to the targets being obscured by other targets, resulting in a large amount of image information being lost.

In summary, it can be seen that the object detection method proposed for low-illumination, blur, dust fog, and dense occluded scenarios can identify targets in these scenarios fast and be highly accurate.

## 5. Conclusions

Aiming to solve the coal flow foreign object detection problem in a coal mine with a low-illumination and dust fog environment, this article proposes a fast and high-accuracy intelligent detection method capable of dealing with multi-layer body occlusion cases for the monitored images of an operating belt conveyor.

(1) For the coal flow foreign object occluded image dataset’s construction, the target occlusion degree, occlusion type, and dense degree are comprehensively considered, and the synthetic occlusion is used. The synthetic occluded images and the real occluded images together constitute the coal flow foreign object occluded image dataset in a low-illumination and dust fog environment.
(2) The YOLOv5 algorithm is improved by the Soft–NMS, SimOTA, Slide Loss, and Inner–SIoU modules. The problem of missed and false detection caused by dense and occluded targets is solved, and the detection accuracy of the model used for small and occluded targets is improved. The mAP_0.5_ of the accuracy improvement model reaches the detection level of YOLOv8s, which is higher than YOLOv5n, YOLOv5s, YOLOv5m, YOLOv7, YOLOv7–X, and YOLOv8n.
(3) The accuracy improvement model is pruned through the Group–Taylor method. After pruning, the mAP_0.5_ of the model is greater than 90%, and its model parameters, FLOPs, and size are also greatly reduced. The detection speed is faster on the GT 1050Ti, GT 1030, and Intel (R) Core (TM) i5–11400F processors, which meets the real-time processing speed requirements.
(4) The mAP_0.5_ of the proposed method on the self-built multi-layer occluded coal flow foreign object dataset is 91.30%, and the mAP_0.5_ is lower than YOLOv5n, YOLOv5s, YOLOv5m, YOLOv7, YOLOv7–X, YOLOv8n, YOLOv8s, and YOLOv8m. However, both the model’s lightweight level and detection speed are superior to the above, and the detection effect of most occluded targets is good in various scenarios.

## Figures and Tables

**Table 1:** Device parameters of image acquisition platform.

Device	Parameters
Belt conveyor	Belt length: 11 m, belt width: 0.8 m, motor frequency range: 0~50 Hz
Belt speed control computer	Operating system: Windows XP, MotorControl software 1.0
Industry camera	Hikvision MV–CA016–10UC–USB3.0 area array camera
Shot	HFO420M, focal length: 4 mm
Camera controller	Operating system: Windows 10, Hikvision client software MVS 4.3.1
Light source	LED strip lamp: 50 cm long, white light source, manually adjusted
Illuminometer	31/2-bit LCD display, measuring range: 0~200,000 Lux, manually adjusted

**Table 2:** Configuration of model training platform.

Platform	Parameters
Operating system	Windows 10
GPU	NVIDIA GeForce RTX 4080, Memory size: 16 GB
CPU	Intel(R) Core(TM)i7–13700KF 3.40GHz
Deep learning framework	Pytorch 2.0.0, CUDA 11.8.0

**Table 3:** Configuration of model speed test platform.

Platform	Parameters
GPU0	NVIDIA GeForce RTX 4080, Pytorch 2.0.0, CUDA 11.8.0
GPU1	GTX 1050Ti, Pytorch 1.10.1, CUDA 10.2.89
GPU2	GT 1030, Pytorch 1.10.1, CUDA 12.2.89
CPU3	Intel(R) Core(TM)i5–11400F 2.60 GHz, Pytorch 1.10.1

**Table 4:** Real and synthetic occluded images without blur and dust fog.

Illumination (Lux)	Real Occlusion Case	Synthetic Occlusion Case
80	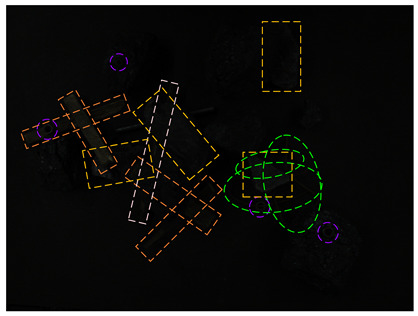	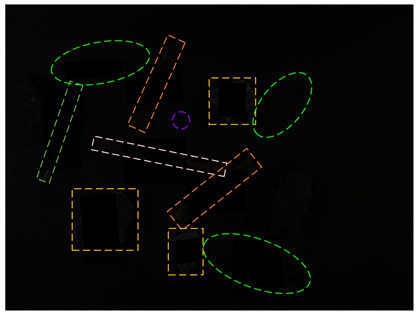
80–V5	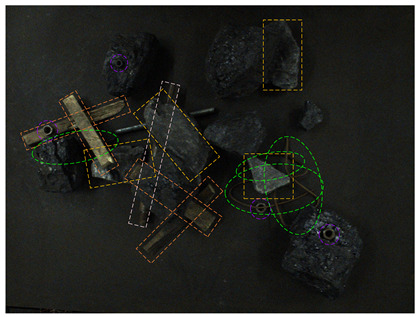	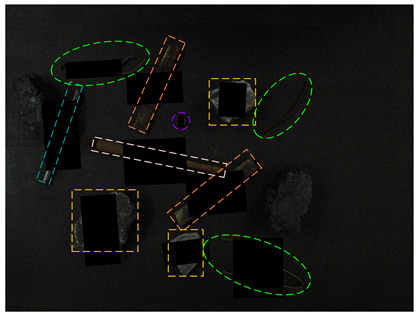
185	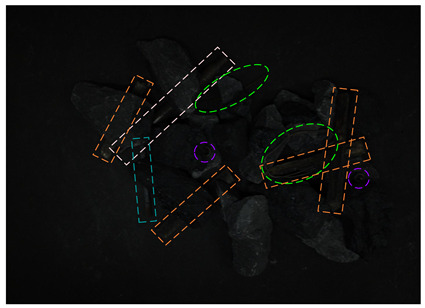	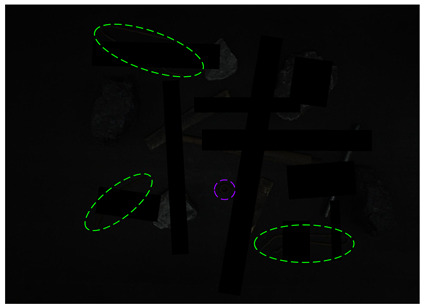
280	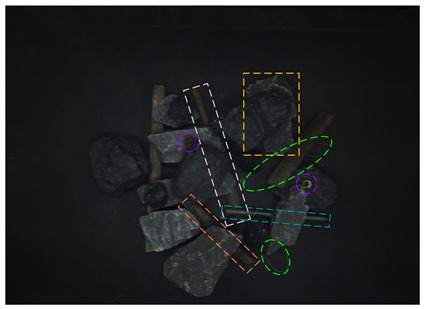	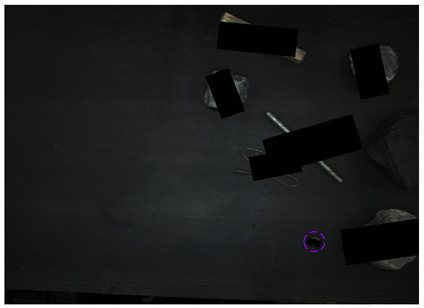

**Table 5:** Real and synthetic occluded images with blur and dust fog.

	Real Occlusion Case	Synthetic Occlusion Case
Blur	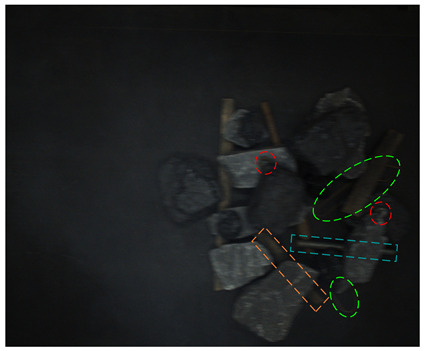	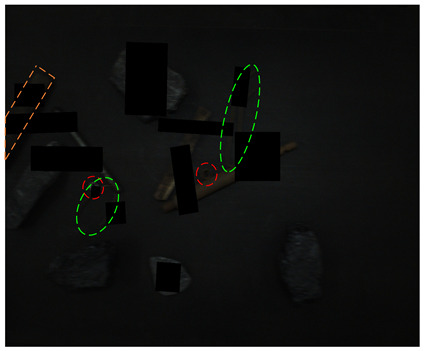
Dust fog	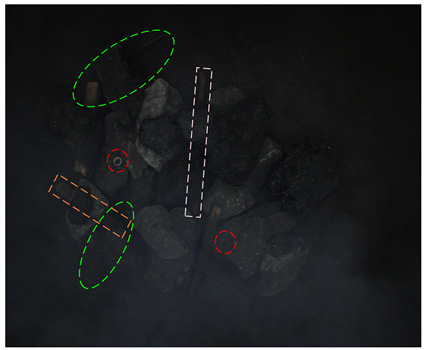	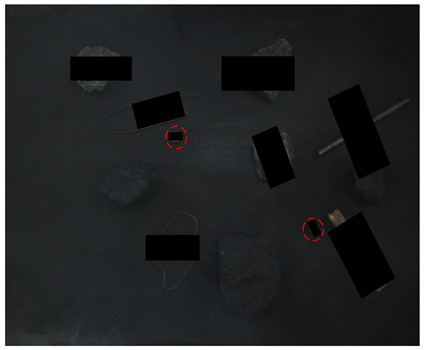
Dust fog–V3	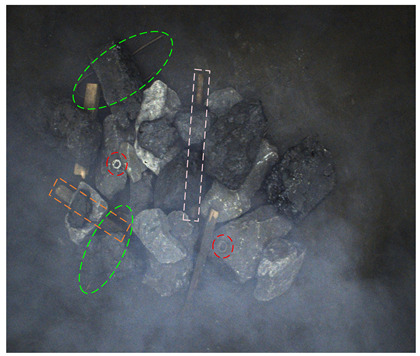	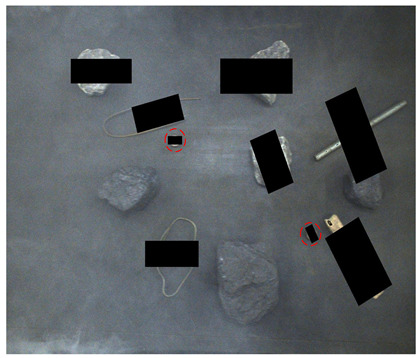

**Table 6:** Results of ablation experiment.

Model	Soft–NMS	SimOTA	Slide Loss	Inner–SIoU	mAP_0.5_/%	mAP_0.75_/%	mAP_0.5:0.95_/%
1					93.9	85.2	74.0
2	√				94.2	88.7	77.5
3	√	√			95.3	90.6	79.6
4	√	√	√		95.9	91.1	79.5
5	√	√	√	√	96.0	91.4	79.8

**Table 7:** Results of model accuracy and complexity for different pruning methods.

Pruning Method	mAP_0.5_/%	mAP_0.75_/%	mAP_0.5:0.95_/%	Parameters /10^5^	FLOPs /10^9^	Model Size /MB	Compression Degree
Model 5	96.00	91.40	79.80	70.26	15.80	14.40	1.00
+Random [34]	90.10	75.60	64.50	4.87	1.20	1.30	14.34
+Lamp [35]	90.30	75.50	64.20	4.87	1.20	1.30	14.34
+L1 [36]	90.00	75.90	64.60	4.20	1.10	1.20	16.74
+Group–Hessian [37]	91.30	76.60	65.30	4.20	1.00	1.20	16.74
+Group–Taylor	91.30	78.20	65.90	4.20	1.00	1.20	16.74

**Table 8:** Results of model detection speed for different pruning methods.

Pruning Method	FPS			
	RTX 4080	GTX 1050Ti	GT 1030	CPU
Model 5	108.03	36.83	14.46	7.63
+Random	105.71	61.93	37.32	29.20
+Lamp	106.36	62.10	38.27	29.60
+L1	104.46	62.79	39.43	32.59
+Group–Hessian	105.82	63.15	39.83	32.61
+Group–Taylor	109.37	66.31	41.90	33.03

**Table 9:** Results of different detection algorithms.

Method	mAP_0.5_/%	Parameters/10^5^	FLOPs/10^9^	Model Size/MB	FPS(CPU)
YOLOv5n	92.50	17.70	4.20	3.90	18.51
YOLOv5n–Group–Taylor	91.00	6.73	1.60	1.70	23.60
YOLOv5s	93.90	70.26	15.80	14.40	7.91
YOLOv5m	94.30	208.73	47.90	42.20	3.78
YOLOv7	94.30	365.07	103.20	74.80	1.65
YOLOv7–X	94.40	708.14	188.10	142.10	1.12
YOLOv8n	95.40	30.07	8.10	6.20	12.67
YOLOv8s	96.00	111.27	28.40	22.50	5.84
YOLOv8m	96.70	258.43	78.70	52.00	2.67
Proposed method	91.30	4.20	1.00	1.20	33.03

## Data Availability

The dataset used in the current study is available from the corresponding author (correspondence: 13323797876@163.com) on reasonable request.
